# Autophagy is Activated *In Vivo* during Trimethyltin-Induced Apoptotic Neurodegeneration: A Study in the Rat Hippocampus

**DOI:** 10.3390/ijms21010175

**Published:** 2019-12-25

**Authors:** Sabrina Ceccariglia, Alessandra Alvino, Aurora Del Fà, Ornella Parolini, Fabrizio Michetti, Carlo Gangitano

**Affiliations:** 1 Dipartimento di Scienze della Vita e Sanità Pubblica, Università Cattolica del Sacro Cuore, 00168 Rome, Italy; sabrina.ceccariglia@unicatt.it (S.C.); alvino.alessandra@gmail.com (A.A.); aurora.delfa@unicatt.it (A.D.F.); ornella.parolini@unicatt.it (O.P.); carloc.gangitano@gmail.com (C.G.); 2 Fondazione Policlinico Universitario A. Gemelli IRCCS, 00168 Rome, Italy; 3 Centro di Ricerca “E. Menni”, Fondazione Poliambulanza—Istituto Ospedaliero, 25124 Brescia, Italy; 4 Dipartimento di Neuroscienze, Università Cattolica del Sacro Cuore, 00168 Rome, Italy; 5 IRCSS Istituto Scientifico San Raffaele, Università Vita-Salute San Raffaele, 20132 Milano MI, Italy

**Keywords:** apoptosis, autophagy, hippocampus, neurodegeneration, Trimethyltin

## Abstract

Trimethyltin (TMT) is an organotin compound known to produce significant and selective neuronal degeneration and reactive astrogliosis in the rodent central nervous system. Autophagy is the main cellular mechanism for degrading and recycling protein aggregates and damaged organelles, which in different stress conditions, such as starvation, generally improves cell survival. Autophagy is documented in several pathologic conditions, including neurodegenerative diseases. This study aimed to investigate the autophagy and apoptosis signaling pathways in hippocampal neurons of TMT-treated (Wistar) rats to explore molecular mechanisms involved in toxicant-induced neuronal injury. The microtubule-associated protein light chain (LC3, autophagosome marker) and sequestosome1 (SQSTM1/p62) (substrate of autophagy-mediated degradation) expressions were examined by Western blotting at different time points after intoxication. The results demonstrate that the LC3 II/I ratio significantly increased at 3 and 5 days, and that p62 levels significantly decreased at 7 and 14 days. Immunofluorescence images of LC3/neuronal nuclear antigen (NeuN) showed numerous strongly positive LC3 neurons throughout the hippocampus at 3 and 5 days. The terminal deoxynucleotidyltransferase dUTP nick end labeling (TUNEL) assay indicated an increase in apoptotic cells starting from 5 days after treatment. In order to clarify apoptotic pathway, immunofluorescence images of apoptosis-inducing factor (AIF)/NeuN did not show nuclear translocation of AIF in neurons. Increased expression of cleaved Caspase-3 was revealed at 5–14 days in all hippocampal regions by Western blotting and immunohistochemistry analyses. These data clearly demonstrate that TMT intoxication induces a marked increase in both autophagy and caspase-dependent apoptosis, and that autophagy occurring just before apoptosis could have a potential role in neuronal loss in this experimental model of neurodegeneration.

## 1. Introduction

TMT is an organotin compound known to produce significant and selective neuronal degeneration in the rodent central nervous system. In particular, it induces a selective loss of pyramidal neurons, especially in the CA1 and CA3/CA4 hippocampal subfields [1,2]. TMT is considered a useful tool to study the molecular mechanisms that occur in the human neurodegenerative diseases, in particular for the hippocampal damage that the neurotoxic triggers, replicating some peculiar features of these processes [3].

Our previous data indicated that, in vitro, the effects of TMT on cultured rat hippocampal neurons are mediated by intracellular calcium homeostasis dysregulation [4]. In vivo, the neuronal cells expressing the Ca^2+^ binding proteins Calretinin and Parvalbumin have been shown to be selectively spared by the neurotoxicant action [5,6,7,8,9], possibly through a calcium-buffering effect that counteracts the TMT-induced calcium overload. Subsequently we demonstrated the involvement of lysosomal Cathepsin D protease during TMT-injury in a calcium-dependent manner [10]. TMT neurodegeneration is also known to be accompanied by astroglial activation [11,12,13,14] and concurrent Aquaporin 4 (AQP4) upregulation in rat hippocampal astrocytes, suggesting the possible involvement of this water channel protein in alterations to vascular permeability and brain edema formation [15]. Although some molecular aspects of action and TMT-induced toxicity have been identified, many other mechanisms remain unclear.

Autophagy is known to be a cellular mechanism for degrading and recycling intracellular proteins and organelles in different physiological and pathological conditions [16,17]. During autophagy activation, cytosolic components are sequestered inside double-membrane vesicles, the autophagosomes, which are conjugated with ubiquitination-linked proteins, the lipidated form of LC3-II and SQSTM1/p62. The formation of autophagosomes is a multistep process that includes phagophore formation, autophagosome elongation/closure, and autolysosome formation that mediates the degradation and recycling of damaged proteins and organelles [18,19].

Autophagy-related (Atg) proteins, a set of evolutionarily conserved proteins, regulate autophagy at different levels. Among these, Beclin-1 (Atg6) is involved in the formation of an initiation complex that promotes autophagosomal membrane nucleation, and the ubiquitin-like conjugation system, Atg12-Atg5, which, together with LC3-II [Atg8 linked to phosphatidylethanolamine (PE)], regulates the autophagosome formation and elongation.

Constitutive autophagy occurs at basal levels and regulates cellular homeostasis, while activated autophagy promotes cell health and survival in response to stressful conditions as starvation, inflammation, hypoxia, and oxidative stress [20,21,22], or paradoxically, it contributes to aggravate cell damage [23,24,25]. In many neurological pathologies, such as cerebral ischemia [26], traumatic brain injury [27,28], and epilepsy [29], autophagy is involved in neuronal injury, but its role is controversial. In fact, in some neurodegeneration models, it contributes to non-apoptotic cell death [30,31,32], while in others it plays a protective role [33,34,35,36].

Previous studies performed in TMT-induced neurodegeneration models showed, using ultrastructural analyses, a formation of autophagic vacuoles in hippocampal neurons after intoxication [37] and indicated that treatment with inducers (as lithium) or inhibitors (as 3-methyladenine) of autophagy, respectively, decreased and aggravated neuronal injury in toxic-treated hippocampal cultures [38]. In addition, impairment of autophagy flux in primary cultures of TMT-treated rat astrocytes has been shown [39].

The present study investigated, for the first time in an animal model, the autophagic process through the analysis of the LC3, p62, and Beclin-1 main regulatory markers, in vivo, in the rat hippocampus at different time points of TMT treatment, in order to understand if autophagy activation or impairment is involved in the neuronal injury. Moreover, while TMT-induced apoptosis in various experimental models has been shown [40,41,42,43,44,45], this study aimed also, through the analysis of the apoptosis molecular signaling pathways, to establish a temporal correlation between autophagy proteins and apoptosis machinery. In this respect, the main apoptosis pathways, such as the classical caspase-dependent mechanisms and the caspase-independent mechanism, involving Apoptosis-Inducing Factor (AIF), have been investigated. Our data showed activation of both autophagy and apoptosis in the rat hippocampus after TMT intoxication. In addition, the expression of the main autophagic and apoptotic markers demonstrated, interestingly, that autophagy is activated just before apoptotic neuronal death, in early treatment times, and continues to remain activated in late times. Since autophagy and apoptosis are involved in numerous human neurodegenerative diseases, this study could offer possibly fertile insight for the analysis of both processes and their mechanistic relation in the pathophysiological aspects of these disorders.

## 2. Results

### 2.1. Autophagy is Activated in the Hippocampus of TMT-Treated Rats

To determine the effects of TMT on autophagy activation in the rat hippocampus, we analyzed the expression of adaptor proteins: LC3 (I and II, respectively the constitutive and active forms) and p62, which conjugates autophagy substrates with LC3 II.

**Figure 1:**
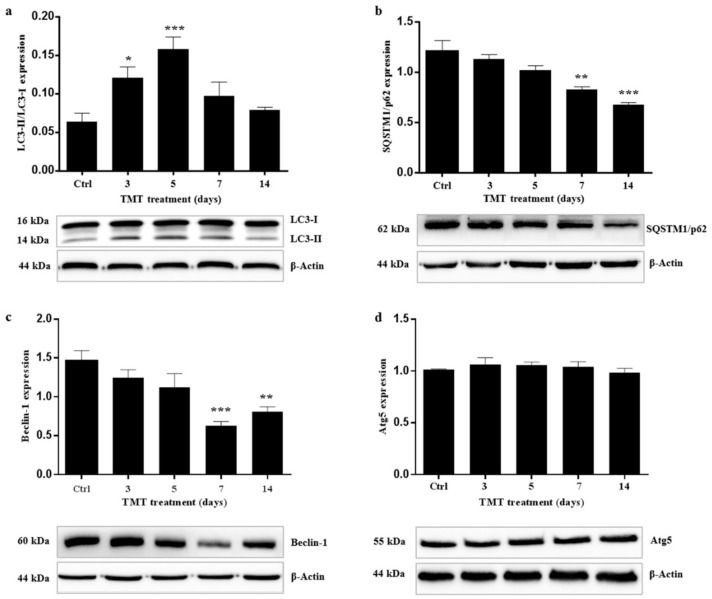
Trimethyltin (TMT) increased LC3II/LC3I, reduced SQSTM1/p62 and Beclin-1 expression levels, and did not affect ATG5 protein in the rat hippocampus after treatment. Graphic presentations and Western blotting images of LC3II/LC3I (**a**), SQSTM1/p62 (**b**), Beclin-1 (**c**), and ATG5 (**d**) proteins are shown. Values are presented as mean ± standard error of the mean (SEM) for each group: the control rats (*n* = 3) and rats treated for 3, 5, 7, and 14 days (*n* = 3/group), * *p* < 0.05, ** *p* < 0.01 and *** *p* < 0.001 compared with controls, Dunnett’s test. Ctrl: control sample.

The conversion of LC3-I to LC3-II (LC3-II/LC3-I) was calculated in order to estimate autophagy flux after Western blot analysis of the single forms (I and II) at 3, 5, 7, and 14 days after treatment. The ratio of LC3II/I increased significantly already from 3 days (*p* < 0.05) after treatment and the peak was observed at 5 days (*p* < 0.001), while from Day 7 it gradually decreased and at 14 days almost returned to the control value (Figure 1a).

**Figure 2:**
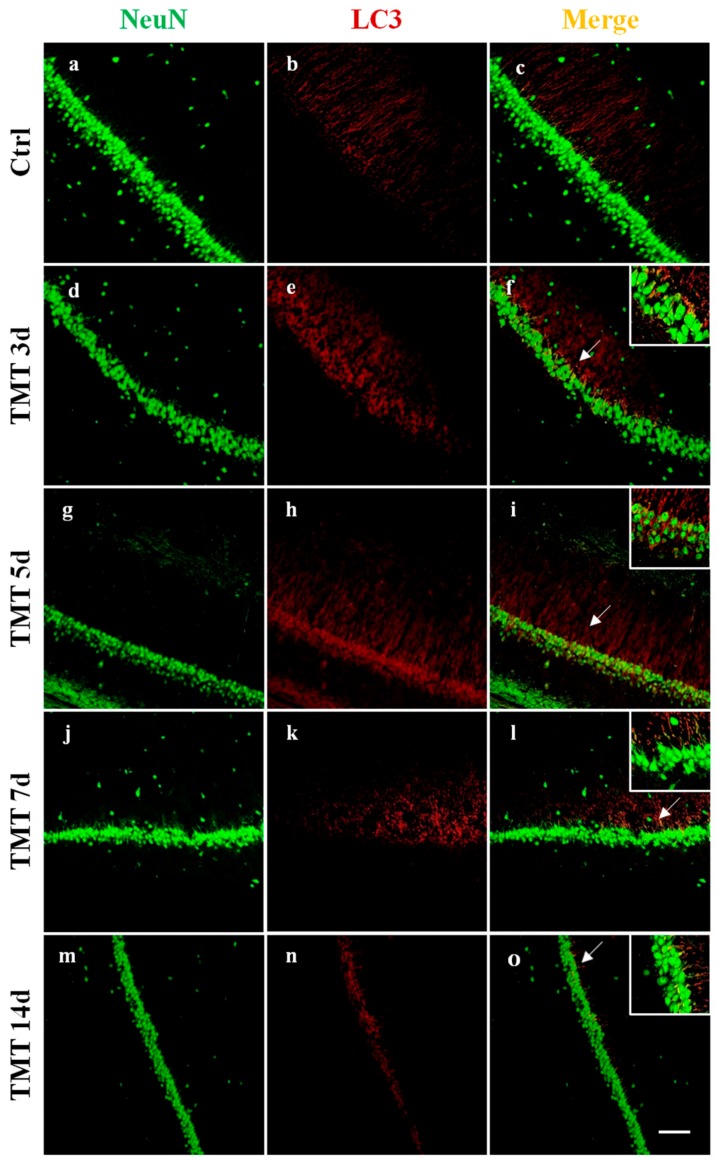
Localization and expression of LC3 in the rat hippocampal neurons, after TMT treatment, in the CA1 area are shown. Sections of the CA1 field at different post-intoxication time-points (3, 5, 7, and 14 days) are labeled for Neu N (green, **a**,**d**,**g**,**j**,**m**), LC3 (red, **b**,**e**,**h**,**k**,**n**), and Neu N/LC3 (merge, **c**,**f**,**i**,**l**,**o**). Control sections (**a**–**c**). At 3 days after treatment, marked LC3 immunoreactivity was observed (**e**) and LC3 labeling strongly increased at 5 days (**h**). LC3 staining decreased at 7 (**k**) and 14 days (**n**) post intoxication. Note the progressive loss of Neu N-positive neurons in TMT-treated rats (**d**,**g**,**j**,**m**) compared with control rats (**a**). Arrows and inserts show details. Scale bar: 100 µm. Ctrl: control sample, d: days. For interpretation of the references to color in this figure legend, the reader is referred to the Web version of this article.

**Figure 3:**
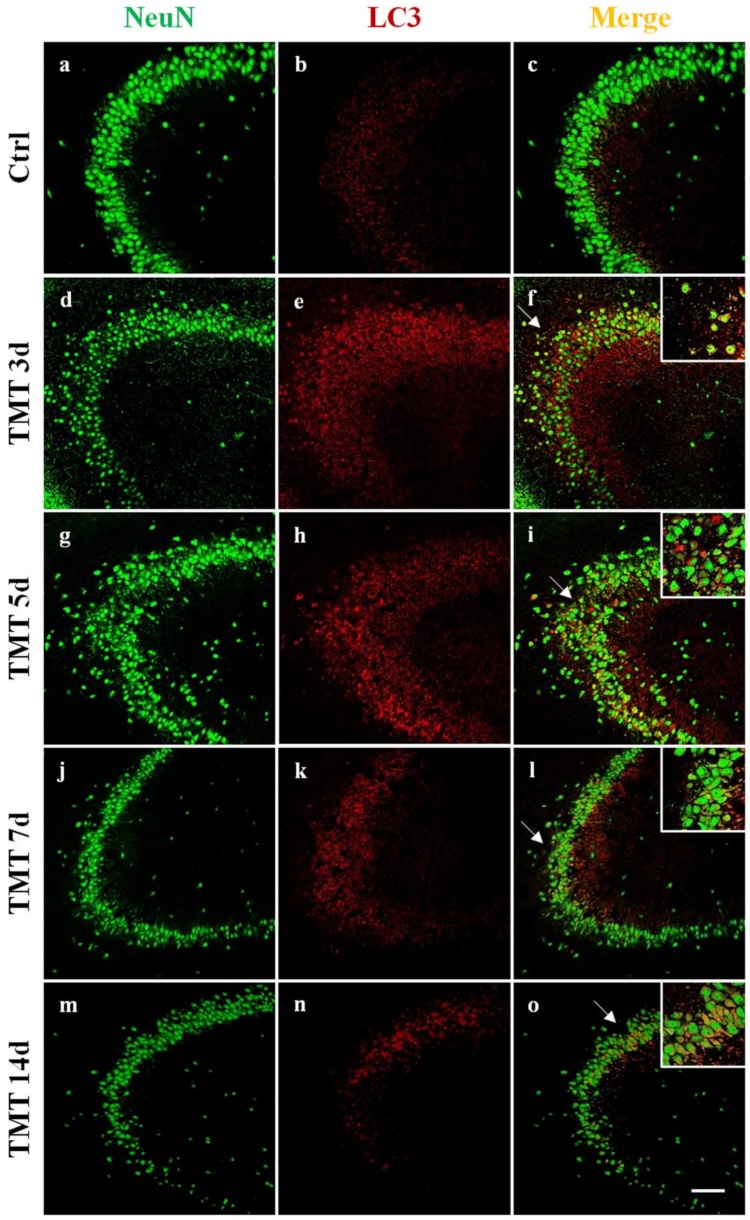
Localization and expression of LC3 in the rat hippocampal neurons, after TMT treatment, in the CA3 area are shown. Sections of the CA3 field at different intoxication time-points (3, 5, 7, and 14 days) are labeled for Neu N (green, **a**,**d**,**g**,**j**,**m**), LC3 (red, **b**,**e**,**h**,**k**,**n**), and Neu N/LC3 (merge, **c**,**f**,**i**,**l**,**o**). Control sections (**a**–**c**). At 3 days after treatment, marked LC3 immunoreactivity was observed (**e**) and LC3 labeling strongly increased at 5 days (**h**). LC3 staining decreased at 7 (**k**) and 14 days (**n**) post intoxication. Note the progressive loss of Neu N-positive neurons in TMT-treated rats (**d**,**g**,**j**,**m**) compared with control rats (**a**). Arrows and inserts show details. Scale bar: 100 µm. Ctrl: control sample, d: days. For interpretation of the references to color in this figure legend, the reader is referred to the Web version of this article.

**Figure 4:**
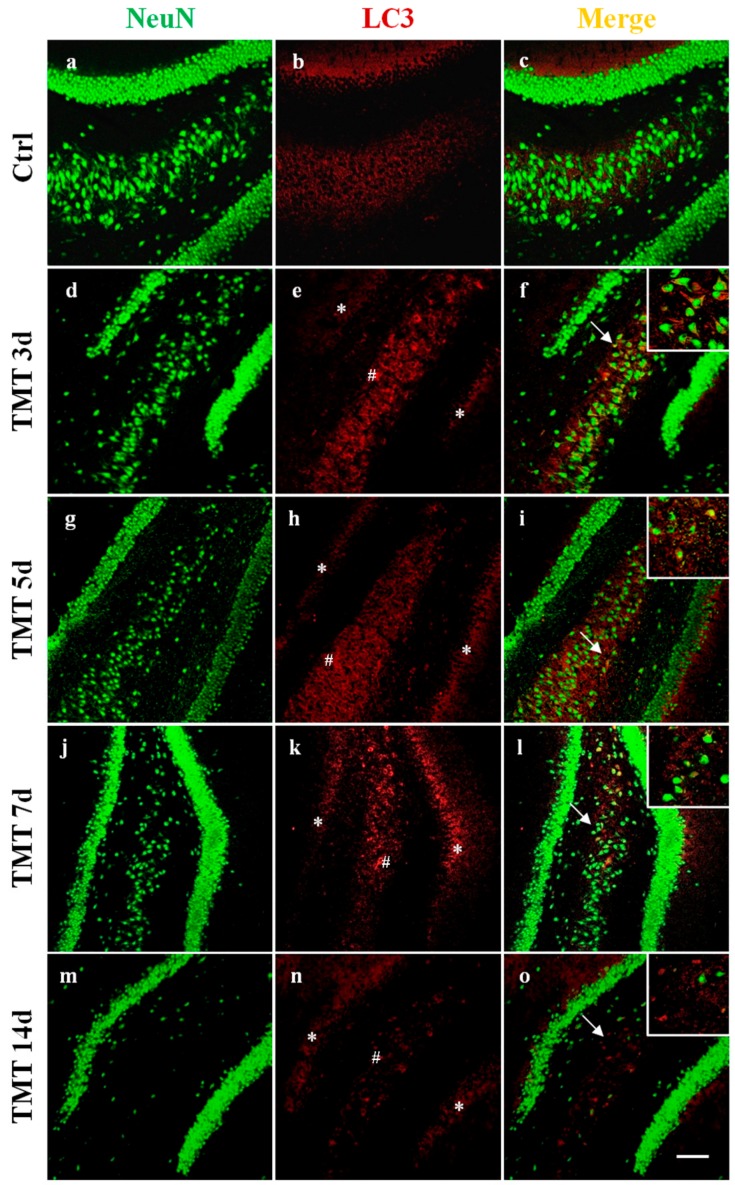
Localization and expression of LC3 in the rat hippocampal neurons, after TMT treatment, in the CA4 area (#) are shown. Sections of the CA4 field at different intoxication time-points (3, 5, 7, and 14 days) are labeled for Neu N (green, **a**,**d**,**g**,**j**,**m**), LC3 (red, **b**,**e**,**h**,**k**,**n**), and Neu N/LC3 (merge, **c**,**f**,**i**,**l**,**o**). Control sections (**a**–**c**). At 3 days after treatment, marked LC3 immunoreactivity was observed (**e**) and LC3 labeling strongly increased at 5 days (**h**). LC3 staining decreased at 7 (**k**) and 14 days (**n**) post intoxication. Non-relevant LC3 reactivity in the Dentate Gyrus (*) was observed at any time of treatment (**e**,**h**,**k**,**n**). Note the progressive loss of Neu N-positive neurons in TMT-treated rats (**d**,**g**,**j**,**m**) compared with control rats (**a**). Arrows and inserts show details. Scale bar: 100 µm. Ctrl: control sample, d: days. For interpretation of the references to color in this figure legend, the reader is referred to the Web version of this article.

In order to visualize the distribution and cellular localization of LC3 in the hippocampus of control and TMT-treated rats, double-labeling immunofluorescence experiments using an antibody to LC3 (which does not distinguish LC3-I from LC3-II) in combination with NeuN marker were performed. We observed a diffuse and marked LC3 immunoreactivity (red) at 3 and 5 days after TMT-intoxication in the CA1 (Figure 2e,h), CA3 (Figure 3e,h), and CA4 areas (Figure 4e,h). LC3 labeling was localized in most NeuN-positive neuronal cells (green) (Figure 2, Figure 3 and Figure 4f,i). At 7 and 14 days, fewer LC3-positive neurons exhibited LC3 reactivity in the same regions (Figure 2, Figure 3 and Figure 4l,o). We have observed, also, a modest LC3 reactivity (red) in the granular neurons of the Dentate Gyrus (green) for all times of treatment (Figure 4f,i,l,o). All experiments performed on the control animal sections evidenced only weak LC3 staining (Figure 2, Figure 3 and Figure 4b) co-localized with NeuN (Figure 2, Figure 3 and Figure 4c).

Interestingly, a progressive loss of NeuN-positive cells in TMT-treated rats (Figure 2, Figure 3 and Figure 4d,g,j,m) compared with control animals (Figure 2, Figure 3 and Figure 4a) was observed.

Western blotting analysis showed levels of p62 protein that gradually decreased starting from 3 days after TMT treatment, with differences that became statistically significant at 7 (*p* < 0.01) and 14 days (*p* < 0.001) compared with the control value (Figure 1b).

### 2.2. TMT Reduces Beclin-1 Levels and Has No Effect on Atg5 Expression

In order to understand better the results of LC3 and p62 expression levels obtained previously and examine how the autophagy system is regulated in the hippocampus in response to TMT-toxicity, we targeted upstream regulatory protein markers Beclin-1 and Atg5. Our results revealed that the levels of Beclin-1 gradually decreased from 3 to 14 days following intoxication, becoming statistically significant at 7 (*p* < 0.001) and 14 days (*p* < 0.01) compared with the control group (Figure 1c). Regarding Atg5, we showed that this protein was not significantly affected by TMT, as its expression levels were similar to those of the controls throughout (Figure 1d).

### 2.3. Apoptotic Cells Increase after TMT-Treatment

**Figure 5:**
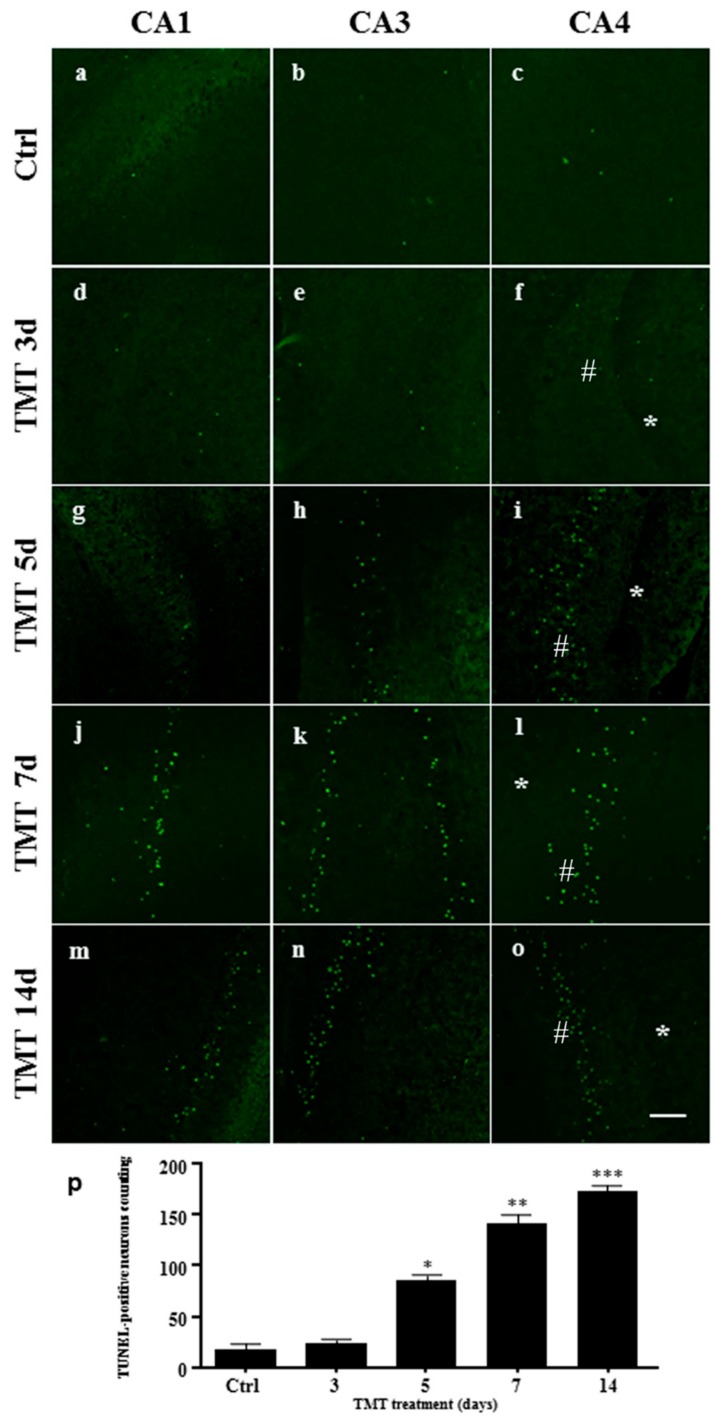
An increased number of apoptotic nuclei in the rat hippocampus after TMT treatment is shown. Representative confocal images of the CA1, CA3, and CA4 (#) areas from the hippocampus of control rats (**a**–**c**) and rats treated with TMT for 3 (**d**–**f**), 5 (**g**–**i**), 7 (**j**–**l**), and 14 (**m**–**o**) days, stained for the presence of apoptotic nuclei (TUNEL staining, green). No apoptotic cells were observed in the Dentate Gyrus (*) at any time of treatment (**f**,**i**,**l**,**o**). Scale bar: 100 µm. Ctrl: control sample, d: days. For interpretation of the references to color in this figure legend, the reader is referred to the Web version of this article. (**p**) The number of TUNEL-positive nuclei. The number of apoptotic nuclei increased gradually and significantly in all the hippocampal areas starting from 5 up to 14 days post treatment. Values are presented as mean±SEM for each group: the control rats (*n* = 3) and 3, 5, 7, and 14 days TMT-treated rats (*n* = 3/group), * *p* < 0.05, ** *p* < 0.01, *** *p* < 0.001 compared with controls, Dunnett’s test.

TUNEL assay was performed to stain DNA strand breaks in the nuclei and examine apoptosis [46] in the hippocampus of the TMT-treated rats. TUNEL-positive nuclei were stained light green, in the CA1, CA3, and CA4 areas, in the control animals and 3, 5, 7, and 14 days after treatment (Figure 5a–o). No TUNEL-positive nuclei were observed in the granular neurons of the Dentate Gyrus in the different times of treatment (Figure 5f,i,l,o). We demonstrated a significant increase in the number of TUNEL-positive nuclei compared with the controls, starting from Day 5 (*p* < 0.05) and gradually increasing between Days 7 (*p* < 0.01) and 14 (*p* < 0.001) after administration of the toxic, when a marked neuronal loss occurred (Figure 5p).

### 2.4. TMT Did Not Induce AIF Nuclear Translocation but Activated Caspase-3 Expression

**Figure 6:**
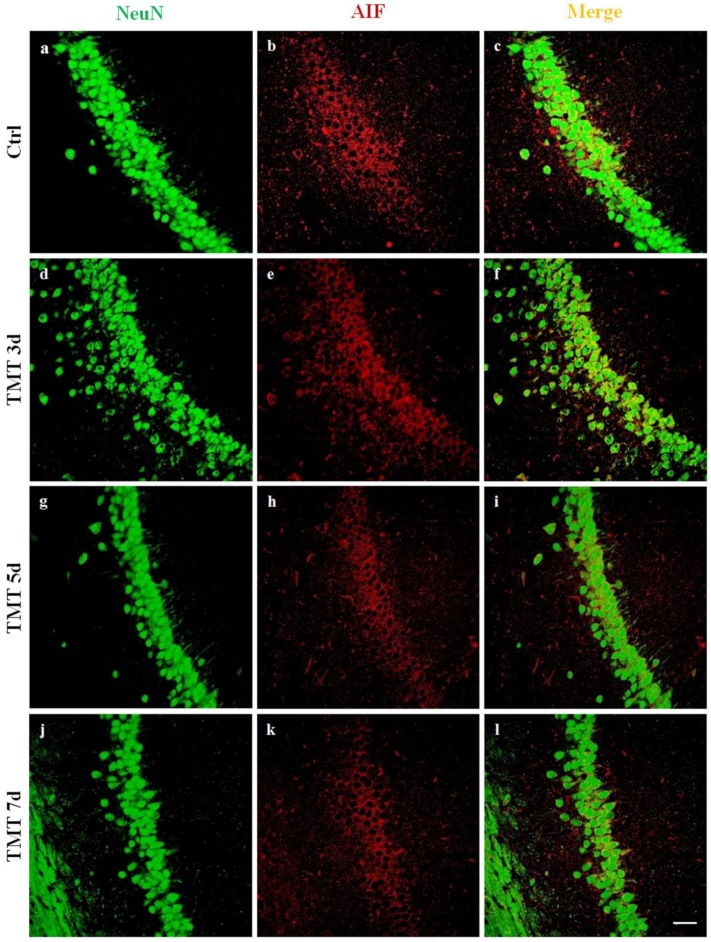
Apoptosis-Inducing Factor (AIF) immunofluorescence labeling in the rat hippocampus after TMT treatment is not localized in the neuron nuclei. Sections of the CA1 field of control (**a**–**c**) and different time-point-treated (3, 5, and 7 days, **d**–**i**) rats, are labeled for Neu N (green, **a**,**d**,**g**,**j**), AIF (red, **b**,**e**,**h**,**k**), and Neu N/AIF (merge, **c**,**f**,**i**,**l**)). AIF did not translocate to the nucleus and is detected exclusively in the cytoplasm of neurons at 3 (**f**), 5 (**i**), and 7 (**l**) days after treatment. Scale bar: 50 µm. Ctrl: control sample, d: days. For interpretation of the references to color in this figure legend, the reader is referred to the Web version of this article.

In order to examine whether TMT induced apoptosis in a Caspase-3-independent manner, we investigated AIF protein translocation from the cytoplasm to the nucleus in rat hippocampal neurons. Double-label immunofluorescence experiments were performed, using an anti-AIF antibody in combination with NeuN neuronal nuclear marker on sections of control rats and rats treated for 3, 5, 7, and 14 days. We observed no localization of AIF in the neuronal nuclei at any time of treatment, while intense immunoreactivity of apoptotic factor AIF (red) co-localized with the cytoplasm of numerous neurons (green) of all hippocampal regions, such as the CA1 (Figure 6), for the different treatment times (3, 5, and 7 days), as shown (Figure 6f,i,l).

No AIF translocation to the nuclei was observed in control sections (Figure 6c).

**Figure 7:**
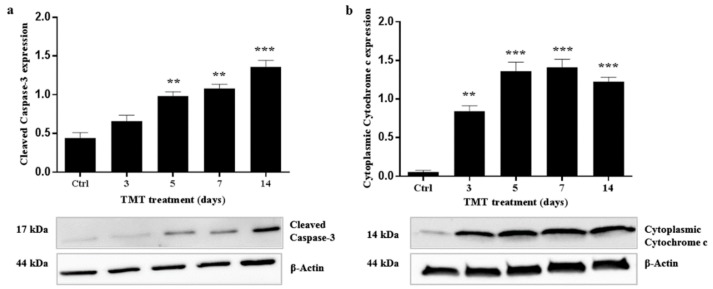
Increased cleaved Caspase-3 and cytoplasmic Cytochrome c expression levels in the rat hippocampus after TMT treatment. Graphic presentations and Western blotting images of cleaved Caspase-3 (**a**) and cytoplasmic Cytochrome c (**b**) proteins are shown. Values are presented as mean±SEM for each group: the control rats (*n* = 3) and rats TMT-treated for 3, 5, 7, and 14 days (*n* = 3/group). ** *p* < 0.01 and *** *p* < 0.001 compared with controls, Dunnett’s-test. Ctrl: control sample.

In order to verify Caspase-3-dependent apoptosis, cleaved Caspase-3 expression was then assessed by investigating the presence of a 17 kDa band corresponding to the active form of the protease at 3, 5, 7, and 14 days after TMT treatment, using Western blot analysis. The cleaved Caspase-3 expression levels were significantly higher than the control value at 5 (*p* < 0.01), 7 (*p* < 0.01), and 14 (*p* < 0.001) days after intoxication (Figure 7a).

**Figure 8:**
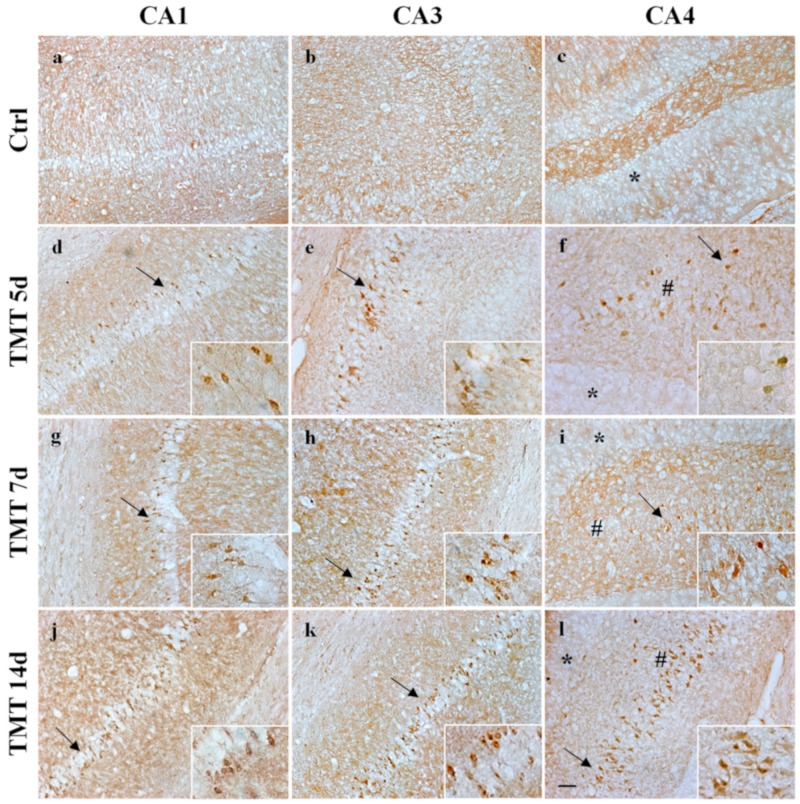
Immunohistochemical labeling of cleaved Caspase-3 in the rat hippocampus after TMT treatment is shown. Cleaved Caspase-3 was detected in brown staining (DAB) at 5 and 7 days in some neurons scattered in the CA1 (**d**,**g**), CA3 (**e**,**h**), and CA4 (# **f**,**i**) areas and in numerous neurons at 14 days after treatment in the same regions (respectively **j**–**l**). No active Caspase-3 immunoreactivity was observed in the control sections of CA1 (**a**), CA3 (**b**), and CA4 (**c**). No Caspase-3 in the granular neurons of the Dentate Gyrus (*) was observed at any time of treatment (**f**,**i**,**l**). Arrows and inserts show details. Scale bar: 50 µm. Ctrl: control sample, d: days. For interpretation of the references to color in this figure legend, the reader is referred to the Web version of this article.

In parallel, we analyzed cleaved Caspase-3 localization in the hippocampus, using DAB-immunohistochemistry experiments. A marked immunoreactivity of cleaved Caspase-3 was observed in the cytoplasm of some cells localized in the CA1, CA3, and CA4 areas at 5 and 7 days (Figure 8d–i) and of numerous cells at 14 days after TMT exposure in the same regions (Figure 8j–l) in accordance with blotting data. Caspase-3-positive cells not only displayed different immunoreactivity levels but also showed evidence of degeneration. We observed no staining for cleaved Caspase-3 at 3 days in any subfields analyzed, probably because our experimental method is not sufficiently sensitive enough to detect low levels (as shows Western blotting analysis) of this protease.

No cleaved Caspase-3 labeling was observed in any of the analyzed hippocampal areas of control rat sections (Figure 8a–c).

### 2.5. Cytoplasmic Expression of Cytochrome c Increases after TMT-Treatment

We detected expression of cytoplasmic Cytochrome c in the rat hippocampus by Western blotting analysis during TMT-induced neurodegeneration because the release of Cytochrome c from the mitochondrial membrane space to the cytosol represents a critical event in activation of the intrinsic apoptotic pathway.

Cytochrome c immunoreactivity was evident as a single 14-kDa band on the cytoplasmic fractions from the hippocampus homogenates at 3, 5, 7, and 14 days after treatment. Cytoplasmic Cytochrome c expression levels showed a gradual and significant increase compared with the control value, starting from 3 (*p* < 0.01) up to 14 days (*p* < 0.001) after exposure to TMT (Figure 7b).

## 3. Discussion

The present study demonstrates, for the first time, that TMT treatment, in vivo, in the rat hippocampus, induces activation of autophagy. The neurotoxicant initially activates autophagy, followed immediately by Caspase-3-dependent apoptosis, as evidenced by the analysis of dynamic changes in the expression of the main molecular markers.

Among the proteins involved in regulation of the autophagic process, LC3 is the most known and studied. LC3 is a soluble protein distributed ubiquitously in mammalian tissues and cultured cells and is considered the major marker of the autophagy pathway. It is cleaved into the LC3-I cytosolic constitutive form and is then conjugated to PE to form active LC3-II. LC3-II is recruited to autophagosomal membranes [47], indicating the number of autophagosome vacuoles formed. In particular, LC3-I is more abundant and readily detected in brain tissue than LC3-II [48]. It is noteworthy in this respect that our double labeling immunofluorescence (LC3/NeuN) images evidenced numerous LC3-positive neurons, likely characterized by an elevated autophagosome number, at 3 and 5 days after TMT treatment, in the CA1, CA3, and CA4 areas (Figure 2, Figure 3 and Figure 4).

We demonstrated that the levels of the LC3-II/LC3-I ratio were significantly increased in the rat hippocampus at 3 and 5 days following intoxication compared with the basal level of the control animals (Figure 1a). Notably, the LC3-II/LC3-I ratio is considered a hallmark of the degree of autophagy activation [49]. These results indicate that TMT action triggers the conversion of LC3-I to LC3-II in the rat hippocampal neurons. Decreased LC3-II/LC3-I values, observed at later treatment times (7 and 14 days) (Figure 1a), suggested both delipidation and recycling of LC3-II to LC3-I (with consequent decrease of LC3-II levels) in the autolysosomal lumen by lysosomal hydrolases [50] and, partly, the effect of a progressive TMT-induced neuronal loss in all the hippocampal regions, especially detected at 14 days. The LC3-II/LC3-I expression levels are in accordance with LC3 reactivity, localized in the hippocampal neurons, which the immunofluorescence images show (Figure 2, Figure 3 and Figure 4).

Changes in the levels of the p62 protein are also used as an indicator of autophagy flux, since p62 is a substrate that is degraded via autophagic pathway [51]. Increased p62 levels that indicate autophagosome-lysosome fusion step impairment (and consequent absence of the cargo degradation) have been observed in some neurodegenerative disorders characterized by accumulation of protein aggregates, including Lewy bodies (in Parkinson’s disease), neurofibrillary tangles (in Alzheimer’s disease), Huntingtin aggregates (in Huntington’s disease), and Mallory bodies (in alcoholic and nonalcoholic steatohepatitis) [52,53,54]. On the contrary, in our model, the results showed a decrease in the p62 values starting from 3 days, which became statistically significant at 7 and 14 compared to the value of the control (Figure 1b), indicating that active autophagosome-lysosome fusion (forming autolysosome) occurs with the consequent degradation of the protein. These data suggest that autophagy is activated by TMT in the hippocampal neurons already at early treatment times.

In order to understand the molecular pathway that regulates autophagy, we also examined the upstream protein Beclin-1. We found that TMT does not stimulate an increase in Beclin-1 expression, but on the contrary induces a gradual decrease of this protein with significant levels at 7 and 14 days (Figure 1c). This result is unexpected. Numerous studies have demonstrated that autophagy activation is generally related to increase of Beclin-1 expression during neurodegeneration process [55,56,57,58]. The decrease of the Beclin-1 levels that we found, however, may merely reflect the involvement, and consequent degradation, of the protein during increased autophagy in our neurodegeneration model. Beclin-1 has also be reported not to be required for the induction of autophagy through the formation of new vacuoles in some experimental models. For example, staurosporine, resveratrol, and H_2_O_2_ have been shown to induce a Beclin-1-independent non-canonical autophagy pathway in different experimental models [59,60,61].

Since examples of Beclin-1-independent but Atg5-dependent autophagy activation have been discussed [59,62], we subsequently explored an autophagy regulation pathway alternative to that of Beclin-1, involving Atg5 protein. Our results showed no significant difference in the levels of Atg5 during the treatment in comparison with the control value (Figure 1d). A change in steady-state levels of Atg5 may therefore not necessarily be required for the induction of autophagy in our model of neurodegeneration.

In this study, we also wanted to further investigate apoptosis triggered by TMT, in order to relate temporally autophagic activity with cellular death, since these processes are often both involved in a wide variety of neurodegenerative diseases. It should be noted that the TMT neurodegeneration shares a pathophysiological characteristic common to other neurodegenerative diseases such as the Alzheimer’s and temporal lobe epilepsy. Thus, TMT-induced neurotoxicity models might be useful tools in researching hippocampal lesions, recovery after brain injury, and specific mechanisms of human neurodegenerative diseases [63].

Much evidence suggests an association between TMT neurodegeneration models and apoptotic neuronal death both in vitro and in vivo. Based on in vitro experiments, Gunasekar and coworkers [41] concluded that low concentrations of TMT (0.01–0.1 µmol/l) cause apoptotic death in cerebellar granule cells. It has also been shown that the incubation with TMT triggers time-/dose-dependent apoptosis in an immortalized hippocampal neuronal cell line (HT-22 cells), which is Caspase-3 mediated [64]. Geloso and collaborators [42] demonstrated, in vivo, that TMT induces apoptotic cell death in adult mice, accompanied by the expression both of cleaved Caspase-3 and of COX-2 in the degenerating hippocampal granular cells.

We showed TUNEL-stained neuronal nuclei to be significantly increased in the CA1, CA3, and CA4 areas at 5 days and, gradually, to peak at 14 days after exposure to the neurotoxicant (Figure 5). In contrast, other studies on rats treated with TMT showed a number of TUNEL-positive neurons in the CA1 and CA3 subfields that peaked in one case at 5 days after TMT gavage (9 mg/kg body weight) [65] and, in another case, at 7 days in the CA3 region after intraperitoneal treatment (7 mg/kg) [66]. This discrepancy could be attributed to the different methods of administration and TMT doses in different experimental settings.

The analysis in time course of the LC3 and p62 expression levels with TUNEL labeling shows a temporal correlation between autophagy and apoptosis activation in TMT-treated rat hippocampal neurons. An autophagic process was activated just before apoptotic neuronal death in early treatment times, and continued to remain activated in late times. On the other hand, we were unable to identify a possible co-localization of the two processes through LC3/TUNEL (or Caspase-3) immunofluorescence double labeling experiments. Further studies, possibly using autophagy inhibitors or inducers, in the TMT-incubated hippocampal cultures, will be needed in order to clarify this aspect.

We also investigated, for the first time, the caspase-independent apoptosis pathway in the TMT-induced neurodegeneration process, where the involvement of the AIF protein has been well established [67,68]. In normal conditions this protein is generally localized in the intermembrane space of mitochondria, where it is involved in oxidative phosphorylation and energy production [69]. In the damaged neurons, it is released to the cytoplasm in response to specific death signals and translocates to the nucleus, where it induces apoptosis [70,71]. The double-label immunofluorescence images for AIF and NeuN showed no nuclear localization of AIF in hippocampal neurons in any of the areas analyzed at the different time points after treatment (Figure 6 for the CA1 area), thus excluding the possible involvement and activation of a caspase-independent apoptotic pathway in the TMT-injured neurons.

We therefore subsequently investigated whether Caspase-3 activation and apoptosis are associated in our experimental system, since this protease is known to be one of the major key regulators of apoptotic mechanisms [72]. Caspases exist as inactive proenzymes and are activated, upon apoptotic signals, after cleavage that can occur via both intrinsic and extrinsic pathways [73,74]. Interestingly, our Western blotting analysis demonstrated increased and significant levels of cleaved Caspase-3 at 5, 7, and 14 days after treatment (Figure 7a). These findings are in accordance with the immunohistochemistry images, which showed numerous cleaved Caspase-3-positive cells (Figure 8) and with TUNEL assay data related to the number of apoptotic nuclei (Figure 5) scattered in all hippocampal subfields at the same treatment times. Importantly, our data showing the expression and localization of Caspase-3 integrate those obtained in a study performed in vivo on TMT-exposed rat hippocampus at early (1–3 days) and late (22–24 days) post-treatment time points, where a marked cleaved Caspase-3 positivity only during the late stages of neuronal damage has been reported using immunohistochemistry [75].

To demonstrate the mitochondrial apoptotic pathway in the present in vivo model, we found the levels of cytoplasmic Cytochrome c to be over-expressed before (3 days) and during (5, 7, and 14 days) Caspase-3 activation (Figure 7b). Cytochrome c is important to maintain cell viability when it is located in the intermembrane space of mitochondria, but when it is released from the damaged mitochondria it activates Caspase-3-dependent cell death [76]. A previous study, in vitro, demonstrated the release of Cytochrome c in the cytosol from damaged mitochondria in primary cultures of hippocampal neurons incubated with TMT [77]. In addition, another earlier study reported that mitochondrial functionality is impaired in PC12 cells after TMT incubation [78]. Evidently, the mitochondrial dysfunction is a prominent phenomenon following TMT treatment. The selective removal of damaged mitochondria through autophagy (indicated as mitophagy) would probably have a great impact in our neurodegeneration model for the protective role against the hippocampal neuron death. Increased accumulation of cytoplasmic Cytochrome c (Figure 7) indicates that, in the hippocampal neurons, there is a mitochondrial dysfunction and that, although there is increased autophagy (Figure 1, Figure 2, Figure 3 and Figure 4), the mitophagy might be defective.

In summary, our study shows for the first time that, in vivo, TMT causes an increase in the LC3 II/LC3 I ratio and a decrease in p62 expression levels in the rat hippocampus, indicating, interestingly, the activation of autophagy during toxic-induced neurodegeneration. We also show that the mitochondrial signaling pathway is involved in TMT-induced apoptosis, including the expression of the Cytochrome c and cleaved Caspase-3. Furthermore, our results document the chronology of the dynamic changes of the main autophagic and apoptotic proteins, evidencing that autophagy is activated just before apoptosis and suggesting a potential role in TMT-induced neuronal loss. One might speculate that, in the early stages of treatment, increased autophagy can represent a protective response of the neurons to the stressful action of TMT. In the later stages, when cellular loss is increased, autophagy can be involved directly in neuronal death or may trigger apoptosis, in the same and/or in different neuron subpopulations. Finally, this study also offers information useful to design new therapeutic strategies based on autophagy modulation in order to prevent or delay neuronal death in the human neurodegenerative disease.

## 4. Materials and Methods

### 4.1. Animal Model

Adult female Wistar rats (200–250 g body weight, age 2 months) were used in this study. The rats were housed in an air-conditioned environment with a constant temperature (25 °C) and had food and water ad libitum on a standardized light/dark cycle. Hippocampal injury was induced by injecting TMT. The rats were divided: in a control group and in groups treated with TMT for 3, 5, 7, and 14 days. The treated rats received a single intraperitoneal injection of TMT (Sigma-Aldrich, St. Louis, MO, USA) (8 mg/kg body weight in a volume of 1 ml/kg body weight, dissolved in saline 0.9% NaCl). The control rats were administered an equal volume of saline solution. Successively, the animals were anesthetized intraperitoneally with 87 mg/kg body weight of Ketavet 100 (Merck Sharp & Dohme, New Jersey, USA) and 12.5 mg/kg body weight of Rompum (Xilazina) (Bayer, Leverkusen, Germany) before being sacrificed. 

The animal protocol (No. 04-01) was reviewed and approved (October 2016) by the Animal Experimentation Committee of Catholic University, Rome. In particular, animal experiments were carried out in accordance with the European Communities Council Directive of 24 November 1986 (86/609/EEC). Every effort was made to minimize the number of animals used and their suffering.

### 4.2. Western Blotting Experiments

Rats (control rats and rats treated for 3, 5, 7, and 14 days, 3 animals/group) were sacrificed by decapitation at various time points of treatment for the Western blotting analyses. The brains were removed, and the hippocampus was rapidly isolated on ice from the ipsilateral hemisphere and stored at −80 °C before being processed. Subsequently, the samples were mechanically homogenized in an ice-cold lysis buffer containing 50 mM Tris–HCL, pH 7.4, 150 mM NaCl, 1% Triton X-100, 0.1% sodium dodecyl sulfate (SDS), 0.5% sodium deoxycholate, and with a protease inhibitor cocktail (Sigma–Aldrich, St. Louis, MO, USA) added just before use. The lysates were incubated on ice for 30 min, sonicated, and then centrifuged at 14,000 *g* for 30 min at 4 °C. The supernatants were used to detect the expression of Beclin-1, Atg5, LC3, p62, and cleaved Caspase-3 proteins.

For Cytochrome c analysis, protein extraction of the cytosolic fractions was performed using a multiple centrifugation method as described elsewhere [65,79]. The hippocampal tissue was mechanically homogenized in a cold suspension buffer [20 mM hydroxyethyl piperazineethanesulfonic acid (HEPES)-KOH, pH 7.5, 250 mM sucrose, 10 mM KCl, 1.5 mM MgCl_2_, 1 mM ethylenediaminetetraacetic acid (EDTA), and 1 mM ethyleneglycotetraacetic acid (EGTA) plus 0.7% protease and phosphatase inhibitor cocktails (Sigma-Aldrich, St. Louis, MO, USA)]. The homogenate was centrifuged at 700 *g* for 10 min at 4 °C and then at 10,000 *g* for 20 min at 4 °C. The supernatant was further centrifuged at 100,000 *g* for 60 min at 4 °C and was then used for cytosolic analysis.

The supernatants were collected and protein concentrations were determined using Bradford’s method [80]. Equal amounts of proteins (60 ug/lane) from each sample were separated by SDS-PAGE in a 12% polyacrylamide gel, blotted at 300 mA for 2 h at 4 °C to Hybond polyvinylidene fluoride (PVDF) membranes (Amersham Biosciences, Uppsala, Sweden) for Beclin-1, Atg5, and p62. LC3 was blotted at 30 mA overnight at 4 °C. For cleaved Caspase-3 and Cytochrome c, respectively, 60 and 40 ug/lane of proteins were electrophoresed in 4–20% precast gel (Biorad, California, USA) and blotted for 7 min using the Trans-Blot Turbo transfer system (Biorad, California, USA). Non-fat dried milk (5% w/v) in Tris–buffered saline (50 mM Tris–HCL, pH 7.4, 150 mM NaCl, 0.1% Tween 20) (TBS-T) was used to block the membranes for 60 min at room temperature (RT). After washing with TBS-T, the membranes were incubated overnight at 4 °C with the appropriate primary antibodies. In particular, rabbit Beclin-1 (1:1000, Cell Signaling Technology, Inc., Leiden, Netherlands), Atg5 (1:1000, Cell Signaling Technology, Inc., Leiden, Netherlands), LC3 (1:1000, Cell Signaling Technology, Inc., Leiden, Netherlands), p62 (1:1000, Cell Signaling Technology, Inc., Leiden, Netherlands), cleaved Caspase-3 (1:1000, Cell Signaling Technology, Inc., Leiden, Netherlands), or Cytochrome c (1:1000, Cell Signaling Technology, Inc., Leiden, Netherlands) and mouse anti-β-Actin (to normalize each protein loading) (1:20,000, Sigma–Aldrich, St. Louis, MO, USA), diluted in 3% non-fat dried milk in TBS-T, were used. After extensive washing with TBS-T, PVDF membranes were incubated for 2 h at RT with adequate horseradish peroxidase-conjugated secondary antibody IgG (1:5000, Invitrogen, CA, USA) diluted in 3% non-fat dried milk in TBS-T. After three washes, bands were visualized using the Chemidoc Imaging System (Biorad, California, USA). Densitometry analysis was performed using ImageJ software.

All Western blotting analyses were performed in at least three independent experiments.

### 4.3. Neuronal Death Assay: TUNEL

The TUNEL assay was performed using a fluorescein in situ cell death detection kit according to the manufacturer’s protocol (In Situ Cell Death Detection Kit, Fluorescein, Roche, Germany). 

The rats (control rats and rats treated for 3, 5, 7, and 14 days, 3 animals/group), after anesthesia, were perfused through the heart with 0.9% saline followed by 4% paraformaldehyde (PFA). The brains were removed, post-fixed in 4% PFA overnight at 4 °C, and cryoprotected in two steps overnight of 20 and 30% sucrose in phosphate buffer saline (PBS) pH 7.4 at 4 °C. Coronal sections were cut on a cryostat (Leica CM 1850) at a thickness of 10 μm. Before the TUNEL assay, the sections were incubated in acetone for 30 min at 4 °C, washed with PBS, placed in 0.5% Triton X-100 for 30 min at RT, and washed three times with PBS. Subsequently, sections were treated with 0.1 M citrate buffer pH 6.0, microwaved for epitope retrieval, and air-cooled. Briefly, 5 μL of enzyme solution (terminal deoxynucleotidyl transferase in storage buffer) were mixed with 45 μL of labeling solution (fluorescein–nucleotide mixture in reaction buffer). Each section was incubated with 50 μL of this TUNEL reaction solution at 37 °C for 60 min.

After further washing with PBS, sections were cover-slipped with a fluoromount aqueous mounting medium (Sigma–Aldrich, St. Louis, MO, USA), examined, and photographed under a Zeiss LSM 510 confocal laser scanning microscopy system.

Finally, we selected the entire hippocampus from each of three non-consecutive brain sections/time point and counted the number of TUNEL-positive neurons.

### 4.4. Immunofluorescence Localization of LC3 and AIF

The rats were sacrificed after anesthesia, and perfused transcardially with 4% PFA, at different time intervals (control and 3, 5, 7, and 14 days after treatment). The brains were removed, post-fixed in 4% PFA overnight at 4 °C, and washed in phosphate buffer (PB), 0.1 M, pH 7.4. Sections were cut on a vibratome (Leica VT 1000S) at a thickness of 40 µm and collected in PB. Nonspecific binding sites were blocked with 5% normal donkey serum in PBS 0.01 M, pH 7.4, for 60 min at RT. Free-floating sections were incubated for double labeling immunofluorescence with primary antibodies—rabbit anti-LC3 (1:100 in PBS) (Cell Signaling Technology, Inc., Leiden, Netherlands) or rabbit anti-AIF (1:200 in PBS) (Santa Cruz Biotechnology, Inc., CA, USA) in combination with mouse anti-NeuN (1:500 in PBS) (Millipore, Temecula, CA, USA) antibody. After rinsing, the primary antibodies were detected by exposing sections to appropriate donkey secondary antibodies—Cy3 anti-rabbit (1:400, Jackson Immunoresearch Laboratories, West Grove, PA, USA) and Cy2 anti-mouse (1:250, Jackson Immunoresearch Laboratories, West Grove, PA, USA)—for 60 min at RT. Finally, sections were mounted on slides, air-dried, and cover-slipped with a fuoromount aqueous medium (Sigma–Aldrich, St. Louis, MO, USA).

Immunofluorescence images were analyzed and photographed under a Zeiss LSM 510 confocal laser scanning microscopy system.

To verify and confirm the specificity of the immunolabeling, primary antisera were omitted, and the sections were incubated only with secondary antibodies. No immunoreactivity was detected.

Five non-consecutive sections were processed for double label immunofluorescence from each animal/time point.

### 4.5. Immunohistochemistry for Cleaved Caspase-3

For cleaved Caspase-3 immunohistochemistry analysis, the brain tissues of rats (control rats and rats treated for 3, 5, 7, and 14 days, 3 animals/group) were processed as described above. Serial coronal sections of 10 μm were cut on a cryostat, mounted on slides, and air-dried. The slides, first, were incubated with acetone for 10 min at −20 °C to enhance antigenicity, and then permeabilized with 0.5% Triton X-100 in PBS overnight at 4 °C. After several rinses in PBS, nonspecific binding sites were blocked for 60 min in PBS containing 5% normal goat serum 0.5% Triton X-100 at RT. Subsequently, the sections were incubated with anti-cleaved Caspase-3 primary antibody (Cell Signaling Technology, Inc., Leiden, Netherlands), diluted 1:250 in PBS-T (PBS with 0.1% Tween 20) containing 5% normal goat serum and 0.5% Triton X-100, on a rotating stand overnight at 4 °C. After washing in PBS, biotinylated goat anti rabbit IgG secondary antibody (Vector Laboratories, Burlingame, CA, USA), diluted 1:200 in PBS and containing 0.3% Triton X-100, was added for 60 min at RT. The sections were then placed in an avidin–biotin peroxidase complex (1:100, Vector Laboratories, Burlingame, CA, USA), rinsed once in PBS, and incubated in a 3,3’-diaminobenzidine (DAB) peroxidase substrate kit as chromogen (Vector Laboratories, Burlingame, CA, USA). Finally, the sections were dehydrated and cover-slipped with a Eukitt mounting medium (Sigma–Aldrich, St. Louis, MO, USA).

Cleaved Caspase-3 analysis was performed using a Zeiss Axiophot microscope, and the images were captured with an AxioCam 503 color camera coupled to the microscope.

The specificity of the labeling was confirmed by incubation of sections with only the secondary antibody, omitting the primary antibody. No immunoreactivity was observed under these conditions.

Five non-consecutive sections were immunostained from each animal/time point.

### 4.6. Statistical Analysis

All statistical analyses were performed using the GraphPad software (GraphPad Prism version 6.01 for Windows, GraphPad Software, La Jolla California USA). The quantitative data were expressed as the mean±SEM. Differences between control groups and TMT-treated rats were assessed using one-way analysis of variance (ANOVA) followed by Dunnett’s multiple comparison post-hoc test, assuming the levels of probability * *p* < 0.05, ** *p* < 0.01, and *** *p* < 0.001 as statistically significant.

## Abbreviations

AIF	Apoptosis-inducing factor
ATG	Autophagy-related
DAB	Diaminobenzidine
GFAP	Glial fibrillary astrocytic protein
LC3	Microtubule-associated protein light chain
NeuN	Neuronal nuclear antigen
PBS	Phosphate buffer saline
PFA	Paraformaldehyde
PVDF	Polyvinylidene fluoride
RT	Room temperature
SDS	Sodium dodecyl sulfate
TBS-T	Tris buffered saline-tween
TMT	Trimethyltin
TUNEL	Terminal deoxynucleotidyltransferase dUTP nick end labeling

## References

[1] Dyer R.S., Walsh T.J., Wonderlin W.F., Bercegeay M. (1982). The trimethyltin syndrome in rats. Neurobehav. Toxicol. Teratol..

[2] Ishida N., Akaike M., Tsutsumi S., Kanai H., Masui A., Sadamatsu M., Kuroda Y., Watanabe Y., McEwen B.S., Kato N. (1997). Trimethyltin syndrome as a hippocampal degeneration model: Temporal changes and neurochemical features of seizure susceptibility and learning impairment. Neuroscience.

[3] Geloso M.C., Corvino V., Michetti F. (2011). Trimethyltin-induced hippocampal degeneration as a tool to investigate neurodegenerative processes. Neurochem. Int..

[4] Piacentini R., Gangitano C., Ceccariglia S., Del Fà A., Azzena G.B., Michetti F., Grassi C. (2008). Dysregulation of intracellular calcium homeostasis is responsible for neuronal death in an experimental model of selective hippocampal degeneration induced by trimethyltin. J. Neurochem..

[5] Geloso M.C., Vinesi P., Michetti F. (1996). Parvalbumin-immunoreactive neurons are not affected by trimethyltin-induced neurodegeneration in the rat hippocampus. Exp. Neurol..

[6] Geloso M.C., Vinesi P., Michetti F. (1997). Calretinin-containing neurons in trimethyltin-induced neurodegeneration in the rat hippocampus: An immunocytochemical study. Exp. Neurol..

[7] Geloso M.C., Vinesi P., Michetti F. (1998). Neuronal subpopulations of developing rat hippocampus containing different calcium-binding proteins behave distinctively in trimethyltin- induced neurodegeneration. Exp. Neurol..

[8] Businaro R., Corvino V., Geloso M.C., De Santis E., Fumagalli L., Michetti F. (2002). De novo expression of calretinin in trimethyltin-induced degeneration of developing rat hippocampus. Mol. Brain Res..

[9] Gangitano C., Falasca C., Del Fà A., Corvino V., Ceccariglia S., Zelano G., Geloso M., Monego G., Michetti F. (2006). Hippocampal calretinin-containing neurons cultured in vitro are resistant to trimethyltin-induced neurodegeneration. Calcium Bind. Proteins.

[10] Ceccariglia S., D’Altocolle A., Del Fa’ A., Pizzolante F., Caccia E., Michetti F., Gangitano C. (2011). Cathepsin D plays a crucial role in the trimethyltin-induced hippocampal neurodegeneration process. Neuroscience.

[11] Fiedorowicz A., Figureiel L., Kaminska B., Zaremba M., Wilk S., Oderfeld-Nowak B. (2001). Dentate granule neuron apoptosis and glia activation in murine hippocampus induced by trimethyltin exposure. Brain Res..

[12] Haga S., Haga C., Aizawa T., Ikeda K. (2002). Neuronal degeneration and glial cell-responses following trimethyltin intoxication in the rat. Acta Neuropathol..

[13] Geloso M.C., Corvino V., Cavallo V., Toesca A., Guadagni E., Passalacqua R., Michetti F. (2004). Expression of astrocytic nestin in the rat hippocampus during trimethyltin-induced neurodegeneration. Neurosci. Lett..

[14] Little A.R., Miller D.B., Li S., Kashon M.L., O’Callaghan J.P. (2012). Trimethyltin-induced neurotoxicity: Gene expression pathway analysis, q-RT-PCR and immunoblotting reveal early effects associated with hippocampal damage and gliosis. Neurotoxicol. Teratol..

[15] Ceccariglia S., D’altocolle A., Del Fa’ A., Silvestrini A., Barba M., Pizzolante F., Repele A., Michetti F., Gangitano C. (2014). Increased expression of Aquaporin 4 in the rat hippocampus and cortex during trimethyltin-induced neurodegeneration. Neuroscience.

[16] Mizushima N. (2007). Autophagy: Process and function. Genes Dev..

[17] Yin Z., Pascual C., Klionsky D.J. (2016). Autophagy: Machinery and regulation. Microb. Cell.

[18] Mizushima N., Yoshimorim T., Levine B. (2010). Methods in Mammalian Autophagy Research. Cell.

[19] Klionsky D.J., Abdelmohsen K., Abe A., Abedin M.J., Abeliovich H., Acevedo Arozena A., Adachi H., Adams C.M., Adams P.D., Adeli K. (2016). Guidelines for the use and interpretation of assays for monitoring autophagy (3rd edition). Autophagy.

[20] Kundu M., Thompson C. (2008). Autophagy: Basic Principles and Relevance to Disease. Annu. Rev. Pathol. Mech. Dis..

[21] Kroemer G., Marino G., Levine B. (2010). Autophagy and the integrated stress response. Mol. Cell.

[22] Noda N.N., Inagaki F. (2015). Mechanisms of Autophagy. Annu. Rev. Biophys..

[23] Shintani T., Klionsky D. (2004). Autophagy in Health and Disease: A Double-Edged Sword. Science.

[24] Levine B., Yuan J. (2005). Autophagy in cell death: An innocent convict?. Rev. Ser..

[25] Sever O., Demir O. (2017). Autophagy: Cell death or survive mechanism. J. Oncol. Sci..

[26] Gabryel B., Kost A., Kasprowska D. (2012). Neuronal autophagy in cerebral ischemia—A potential target for neuroprotective strategies?. Pharmacol. Rep..

[27] Chen S., Atkins C.M., Liu C.L., Alonso O.F., Dietrich W.D., Hu B.R. (2007). Alterations in mammalian target of rapamycin signaling pathways after traumatic brain injury. J. Cereb. Blood Flow Metab..

[28] Liu C.L., Chen S., Dietrich D., Hu B.R. (2008). Changes in autophagy after traumatic brain injury. J. Cereb. Blood Flow Metab..

[29] Li Q., Han Y., Du J., Jin H., Zhang J., Niu M., Qin J. (2018). Alterations of apoptosis and autophagy in developing brain of rats with epilepsy: Changes in LC3, P62, Beclin-l and Bcl2 levels. Neurosci. Res..

[30] Koike M., Shibata M., Tadakoshi M., Gotoh K., Komatsu M., Waguri S., Kawahara N., Kuida K., Nagata S., Kominami E. (2008). Inhibition of autophagy prevents hippocampal pyramidal neuron death after hypoxic-ischemic injury. Am. J. Pathol..

[31] Ginet V., Spiehlmann A., Rummel C., Rudinskiy N., Grishchuk Y., Luthi-Carter R., Clarke P.G.H., Truttmann A.C., Puyal J. (2014). Involvement of autophagy in hypoxic excitotokic.neuronal death. Autophagy.

[32] Descloux C., Ginet V., Rummel C., Truttmann A.C., Puyal J. (2018). Enhanced autophagy contributes to excitotoxic lesions in a rat model of preterm brain injury. Cell Death Dis..

[33] Spencer B., Potkar R., Trejo M., Rockenstein E., Gindi R., Adame A., Wyss-coray T., Masliah E. (2009). Beclin 1 gene transfer activates autophagy and ameliorates the neurodegenerative pathology in α-synuclein models of parkinson’s and lewy body disease. J. Neurosci..

[34] Lee J., Yu W.H., Kumar A., Lee S., Mohan P.S., Peterhoff C.M., Wolfe D.M., Martinez-Vicente M., Massey A.C., Uchiyama Y. (2010). Lysosomal Proteolysis and Autophagy Require Presenilin 1 and Are Disrupted by Alzheimer-Related PSI Mutations. Cell.

[35] Hochfeld W.E., Lee S., Rubinsztein D.C. (2013). Therapeutic induction of autophagy to modulate neurodegenerative disease progression. Acta Pharmacol. Sin..

[36] Ruffoli R., Bartalucci A., Frati A., Fornai F. (2015). Ultrastructural studies of ALS mitochondria connect altered function and permeability with defects of mitophagy and mitochondriogenesis. Front. Cell Neurosci..

[37] Bouldin T.W., Goines N.D., Bagnell R.C., Krigman M.R. (1981). Pathogenesis of trimethyltin neuronal toxicity. Ultrastructural and cytochemical observations. Am. J. Pathol..

[38] Fabrizi C., Somma F., Pompili E., Biagioni F., Lenzi P., Fornai F., Fumagalli L. (2012). Role of autophagy inhibitors and inducers in modulating the toxicity of trimethyltin in neuronal cell cultures. J. Neural Transm..

[39] Fabrizi C., Pompili E., De Vito S., Somma F., Catizone A., Ricci G., Lenzi P., Fornai F., Fumagalli L. (2016). Impairment of the autophagic flux in astrocytes intoxicated by trimethyltin. Neurotoxicology.

[40] Kane M.D., Yang C.W., Gunasekar P.G., Isom G.E. (1998). Trimethyltin Stimulates Protein Kinase C Translocation Through Receptor-Mediated Phospholipase C Activation in PC12 Cells. J. Neurochem..

[41] Gunasekar P., Li L., Prabhakaran K., Eybl V., Borowitz J., Isom G. (2001). Mechanisms of the apoptotic and necrotic actions of trimethyltin in cerebellar granule cells. Toxicol. Sci..

[42] Geloso M.C., Vercelli A., Corvino V., Repici M., Boca M., Haglid K., Zelano G., Michetti F. (2002). Cyclooxygenase-2 and caspase 3 expression in trimethyltin-induced apoptosis in the mouse hippocampus. Exp. Neurol..

[43] Jenkins S.M., Barone S. (2004). The neurotoxicant trimethyltin induces apoptosis via caspase activation, p38 protein kinase, and oxidative stress in PC 12 cells. Toxicol. Lett..

[44] Buck-Koehntop B.A., Mascioni A., Buffy J.J., Veglia G. (2005). Structure, dynamics, and membrane topology of stannin: A mediator of neuronal cell apoptosis induced by trimethyltin chloride. J. Mol. Biol..

[45] Yuliani S., Widyarini S., Mustofa, Partadiredja G. (2017). Turmeric extract inhibits apoptosis of hippocampal neurons of trimethyltin-exposed rats. Bratisl. Med. J..

[46] Gavrieli Y., Sherman Y., Ben-Sasson S.A. (1992). Identification of programmed cell death in situ via specific labeling of nuclear DNA fragmentation. J. Cell Biol..

[47] Tanida I., Ueno T., Kominami E. (2004). LC3 conjugation system in mammalian autophagy. Int. J. Biochem. Cell Biol..

[48] Yang D.S., Stavrides P., Mohan P.S., Kaushik S., Kumar A., Ohno M., Schmidt S.D., Wesson D., Bandyopadhyay U., Jiang Y. (2011). Reversal of autophagy dysfunction in the TgCRND8 mouse model of Alzheimer’s disease ameliorates amyloid pathologies and memory deficits. Brain.

[49] Klionsky D., Abeliovich A., Agostinis P., Al E. (2008). Guidelines for the use and interpretation of assays for monitoring autophagy in higher eukaryotes. Autophagy.

[50] Mizushima N., Yoshimori T. (2007). How to interpret LC3 immunoblotting. Autophagy.

[51] Katsuragi Y., Ichimura Y., Komatsu M. (2015). P62/SQSTM1 functions as a signaling hub and an autophagy adaptor. FEBS J..

[52] Knævelsrud H., Simonsen A. (2010). Figurehting disease by selective autophagy of aggregate-prone proteins. FEBS Lett..

[53] Komatsu M., Ichimura Y. (2010). Physiological significance of selective degradation of p62 by autophagy. FEBS Lett..

[54] Rogov V., Dötsch V., Johansen T., Kirkin V. (2014). Interactions between Autophagy Receptors and Ubiquitin-like Proteins Form the Molecular Basis for Selective Autophagy. Mol. Cell.

[55] Zhang Y.B., Li S.X., Chen X.P., Yang L., Zhang Y.G., Liu R., Tao L.Y. (2008). Autophagy is activated and might protect neurons from degeneration after traumatic brain injury. Neurosci. Bull..

[56] Luo C.L., Li B.X., Li Q.Q., Chen X.P., Sun Y.X., Bao H.J., Dai D.K., Shen Y.W., Xu H.F., Ni H. (2011). Autophagy is involved in traumatic brain injury-induced cell death and contributes to functional outcome deficits in mice. Neuroscience.

[57] Shi R., Weng J., Zhao L., Li X.M., Gao T.M., Kong J. (2012). Excessive Autophagy Contributes to Neuron Death in Cerebral Ischemia. CNS Neurosci. Ther..

[58] Guo D., Ma J., Yan L., Li T., Li Z., Han X., Shui S. (2017). Down-Regulation of Lncrna MALATI Attenuates Neuronal Cell Death Through Suppressing Beclin l-Dependent Autophagy by Regulating Mir-30a in Cerebral Ischemic Stroke. Cell. Physiol. Biochem..

[59] Grishchuk Y., Ginet V., Truttmann A.C., Clarke P.G.H., Puyal J. (2011). Beclin I-independent autophagy contributes to apoptosis in cortical neurons. Autophagy.

[60] Mauthe M., Jacob A., Freiberger S., Hentschel K., Stierhof Y.D., Codogno P., Proikas-Cezanne T. (2011). Resveratrol-mediated autophagy requires WIPI-1-regulated LC3 lipidation in the absence of induced phagophore formation. Autophagy.

[61] Seo G., Kim S.K., Byun Y.J., Oh E., Jeong S.W., Chae G.T., Lee S.B. (2011). Hydrogen peroxide induces Beclin 1-independent autophagic cell death by suppressing the mTOR pathway via promoting the ubiquitination and degradation of Rheb in GSH-depleted RAW 264.7 cells. Free Rad. Res..

[62] Zhu J.H., Horbinski C., Guo F., Watkins S., Uchiyama Y., Chu C.T. (2007). Regulation of autophagy by extracellular signal-regulated protein kinases during 1-methyl-4-phenylpyridinium-induced cell death. Am. J. Pathol..

[63] Lee S., Yang M., Kim J., Kang S., Kim J., Kim J.C., Jung C., Shin T., Kim S.H., Moon C. (2016). Trimethyltin-induced hippocampal neurodegeneration: A mechanism-based review. Brain Res. Bull..

[64] Zhang L., Li L., Prabhakaran K., Borowitz J.L., Isom G.E. (2006). Trimethyltin-induced apoptosis is associated with upregulation of inducible nitric oxide synthase and Bax in a hippocampal cell line. Toxicol. Appl. Pharmacol..

[65] Morita M., Imai H., Liu Y., Xu X., Sadamatsu M., Nakagami R., Shirakawa T., Nakano K., Kita Y., Yoshida K. (2008). FK506-protective effects against trimethyltin neurotoxicity in rats: Hippocampal expression analyses reveal the involvement of periarterial osteopontin. Neuroscience.

[66] Nilsberth C., Kostyszyn B., Luthman J. (2002). Changes in APP, PS1 and other factors related to Alzheimer’s disease pathophysiology after trimethyltin-induced brain lesion in the rat. Neurotox. Res..

[67] Joza N., Susin S.A., Daugas E., Stanford W.L., Cho S.K., Li C.Y.J., Sasaki T., Elia A., Al E. (2001). Essential role of the mitochondrial apoptosis-inducing factor in programmed cell death. Nature.

[68] Krantic S., Mechawar N., Reix S., Quirion R. (2007). Apoptosis-inducing factor: A matter of neuron life and death. Prog. Neurobiol..

[69] Vahsen N., Candé C., Brière J.J., Bénit P., Joza N., Larochette N., Mastroberardino P.G., Pequignot M.O., Casares N., Lazar V. (2004). AIF deficiency compromises oxidative phosphorylation. EMBO J..

[70] Susin S.A., Daugas E., Ravagnan L., Samejima K., Zamzami N., Loeffler M., Costantini P., Ferri K.F., Irinopoulou T., Prévost M.-C. (2000). Two Distinct Pathways Leading to Nuclear Apoptosis. J. Exp. Med..

[71] Otera H., Ohsakaya S., Nagaura Z.I., Ishihara N., Mihara K. (2005). Export of mitochondrial AIF in response to proapoptotic stimuli depends on processing at the intermembrane space. EMBO J..

[72] Cohen G.M. (1997). Caspases: The executioners of apoptosis. Biochem. J..

[73] Mignotte B., Vayssiere J. (1998). Review Mitochondria and apoptosis. Eur. J. Biochem..

[74] Porter A.G., Janicke R.U. (1999). Emerging roles of Caspase-3 in apoptosis. Cell Death Differ..

[75] Gasparova Z., Janega P., Stara V., Ujhazy E. (2012). Early and late stage of neurodegeneration induced by trimethyltin in hippocampus and cortex of male Wistar rats. Neuro Endocrinol. Lett..

[76] Deniaud A., Sharaf El Dein O., Maillier E., Poncet D., Kroemer G., Lemaire C., Brenner C. (2008). Endoplasmic reticulum stress induces calcium-dependent permeability transition, mitochondrial outer membrane permeabilization and apoptosis. Oncogene.

[77] Qu M., Zhou Z., Chen C., Li M., Pei L., Chu F., Yang J., Wang Y., Li L., Liu C. (2011). Lycopene protects against trimethyltin-induced neurotoxicity in primary cultured rat hippocampal neurons by inhibiting the mitochondrial apoptotic pathway. Neurochem. Int..

[78] Misiti F., Orsini F., Clementi M.E., Lattanzi W., Giardina B., Michetti F. (2008). Mitochondrial oxygen consumption inhibition importance for TMT-dependent cell death in undifferentiated PC12 cells. Neurochem. Int..

[79] Morita-Fujimura Y., Fujimura M., Kawase M., Chen S.F., Chan P.H. (1999). Release of mitochondrial cytochrome c and DNA fragmentation after cold injury-induced brain trauma in mice: Possible role in neuronal apoptosis. Neurosci. Lett..

[80] Bradford M. (1976). A rapid and sensitive method for the quantitation of microgram quantities of protein utilizing the principle of protein-dye binding. Anal. Biochem..

